# Outcomes in Elderly Patients with Glioblastoma Multiforme Treated with Short-Course Radiation Alone Compared to Short-Course Radiation and Concurrent and Adjuvant Temozolomide Based on Performance Status and Extent of Resection

**DOI:** 10.3390/curroncol28040220

**Published:** 2021-06-26

**Authors:** Taskia Mir, Gregory Pond, Jeffrey N. Greenspoon

**Affiliations:** Juravinski Cancer Center, Department of Oncology, Radiation Oncology, McMaster University, 699 Concession St., Hamilton, ON L8V5C2, Canada; gpond@mcmaster.ca (G.P.); greenspj@hhsc.ca (J.N.G.)

**Keywords:** GBM, KPS, elderly, treatment outcomes, toxicity

## Abstract

(1) Background: Studies in elderly patients over the age of 65 with glioblastoma have shown survival benefits of short-course radiation therapy with concurrent and adjuvant temozolomide, making it the standard of care adopted at Juravinski Cancer Center. Our study retrospectively examines patients with GBM aged ≥ 70 at the JCC treated with short-course radiation alone compared to those treated with short-course radiation and concurrent and adjuvant TMZ, to determine if there is a difference in outcomes based on performance status. (2) Methods: A retrospective chart review was conducted at JCC using patients diagnosed with GBM in 2014–2017 (treated with the old protocol of short-course RT alone) versus those diagnosed in 2017–2019 (treated with the new protocol of short-course radiation and TMZ). Patient demographics, treatments, outcomes, and baseline KPS were analyzed. (3) Results: No clear benefit and more neurologic decline post treatment were seen in patients with borderline performance status and subtotal resection who underwent concurrent treatment with temozolomide and radiation. The addition of temozolomide was most helpful in patients with good performance status and a gross total resection. Variable outcomes were seen in patients with mixed traits. (4) Conclusions: This study suggests that performance status and extent of resection are significant determinants of patient response to treatment. In the case of elderly patients with borderline performance status and GTR or those with good performance status and STR, also described as “mixed traits”, it may be beneficial to pursue single modality treatment, ideally based on MGMT promoter methylation status as opposed to bimodality treatment in order to maintain the best QOL.

## 1. Introduction

Glioblastoma (GBM) constitutes 54% of all gliomas and 16% of all new adult primary brain tumors diagnosed every year [1]. The median survival with GBM is less than two years, even with combined modality treatment [2]. Traditionally, the treatment of GBM included only surgical resection and radiation (6000 cGy in 30 fractions). In 2005, the treatment paradigm for GBM was changed as a result of the trial by Stupp et al. [2]. Treatment of GBM with surgical gross total resection followed by concurrent and adjuvant radiation and temozolomide (TMZ) chemotherapy was established as the standard of care in patients aged 65 and younger [2]. In this population, overall survival has improved from 8 to 10 months to 12 to 14 months with the addition of TMZ [2]. As the population ages, however, the incidence of GBM has increased among elderly patients [3]. Most research on GBM including the Stupp et al. trial has been conducted using patients with an average age of 55, whereas the population-based median age of patients with GBM is 65 [1].

Management of GBM in elderly population can be challenging given the higher rate of toxicity with standard treatment, as well as increased pre-existing medical comorbidities, making it difficult for patients to tolerate and recover from side effects of treatment [3]. Studies have shown that short-course radiation provides the same expected survival as standard radiation in elderly patients [4]. The previous standard of care was to treat elderly patients only with surgical resection as appropriate followed by short-course radiation (4005 cGy in 15 fractions). A 2017 study by Perry et al. compared clinical outcomes of patients aged 65 and above who were treated with short-course radiation therapy alone with those who were treated with short-course radiation and adjuvant and concurrent TMZ [5]. This study included all patients with ECOG 0–2. This translates to patients with KPS ≥ 50. Results of this study showed an improvement in overall survival from 7.6 months to 9.3 months in patients treated with the addition of TMZ. As a result, this has become the standard of care adopted widely across the country unless the patient has major contraindications. If the tumor is too large for surgery or the tumor volume is felt to be too large for save radiation in someone with poor clinical status and they are known to have a MGMT promoter methylated tumor, they may be offered TMZ alone.

In practice, some elderly patients with GBM at the Juravinski Cancer Center (JCC) struggled to tolerate concurrent radiation and chemotherapy. It was felt by the treatment group at the JCC that this morbidity is associated with patients who have a borderline performance status (KPS ≤ 80). The morbidity resulting from this treatment may be affecting their overall survival and quality of life. In particular, neurologic decline and severe fatigue are known side effects of treatment with TMZ and radiation [6].

Our study uses a retrospective chart review to examine clinical outcomes of patients with GBM aged 70 and above at the JCC treated between 2014 and 2017, when radiation monotherapy was the institutional standard, compared to those treated between 2017 and 2019, after the addition of TMZ to our institutional standard protocol. We categorized these patients based on their pretreatment Karnofsky performance status (KPS) to compare the overall survival (OS) and rates of toxicity between the two groups. We also evaluated the rates of thrombocytopenia and neurologic decline post treatment in these patients to assess potential adverse effects of treatment with combined radiation and TMZ. We recorded overall survival in our observed group and compared this to the previously reported overall survival [5]. We subcategorized the patients to those who had a gross total resection (>95%), subtotal resection (50–95%), and biopsy only (<50%) in order to record survival outcomes in these patients, as the extent of resection and KPS are the most important factors in determining survival [7].

## 2. Methods

We conducted a retrospective chart review at JCC using patients diagnosed with GBM between December 2013 and June 2016 (treated with short-course RT alone) compared to patients diagnosed with GBM between June 2016 and October 2019 (treated with short-course radiation and concurrent and adjuvant TMZ). Patients were followed up to February 2020. Patients treated outside of standard protocol were excluded, such as those treated with TMZ alone, as they were likely to have other medical comorbidities confounding their management. Patient demographics (age, comorbidities, and gender) were recorded. Patient baseline KPS was recorded either using information standardly recorded in our clinics or was inferred from clinical notes when not available. The location of the tumor was recorded based on CT, MRI, and clinical notes (Table S1). The extent of resection was determined using MRI and surgical notes. Patients were divided between those who had gross total resection (≥95%) and those who had subtotal resection/biopsy (<95%). The extent of initial surgical resection was recorded. Using CTCAE, neurologic toxicity was defined as any nervous system disorder that developed following treatment [8]. In order to help with analysis in this retrospective study, we focused specifically on any neurologic decline following therapy as seen in the clinical notes. We divided patients into those with KPS < 80 (borderline performance status) and those with KPS ≥80 (good performance status) and compared these patients to determine if there is a difference between the observed and study-recorded OS.

Descriptive statistics were used to summarize patient characteristics and outcomes. Chi-square and Wilcoxon rank-sum tests were used to compare patient characteristics between cohorts. The Kaplan–Meier method was used to estimate overall (OS) and progression-free (PFS) survival, and a comparison between the radiation alone and chemoradiation groups was performed using the log-rank test. Cox regression was used to investigate prognostic factors of OS and PFS; the results of OS and PFS were very similar, hence, only OS results are presented for brevity. OS was measured from the date of diagnosis until the date of death. Patients without a known death date were censored on the last date patients were confirmed to be alive. A Fisher’s exact test was used to compare the rates of thrombocytopenia amongst patients who received TMZ at any time with those who did not. All tests were two-sided and a *p*-value of <0.05 was considered statistically significant.

## 3. Results

The demographic data is shown in Table 1. A total of 80 elderly patients treated at the JCC for GBM were reviewed between 2014 and 2019. Of those, 54% were male and 46% were female. The median age of these patients was 73 with a range of 65 to 86. In total, 32 patients had a KPS > 80 (good performance status), while 48 patients had a KPS < 80 (borderline performance status). The range of KPS in treated patients was 40–90. The mean baseline MMSE of treated patients was 27. All patients were intended to receive 40 Gy in 15 fractions of radiation and only one patient did not complete radiation treatment as intended. Of the 80 patients examined, 47 received concurrent TMZ and only 26 of those 47 patients received adjuvant TMZ. In a majority of these cases, patients did not go on to have adjuvant TMZ due to poor performance or toxicity.

Based on the post-operative MRI, 19 patients were initially treated with a gross total resection of their tumor (>95%). Additionally, 40 patients were initially treated with a subtotal resection (50–95) and 21 patients received a biopsy only (≤50%).

**Figure 1:**
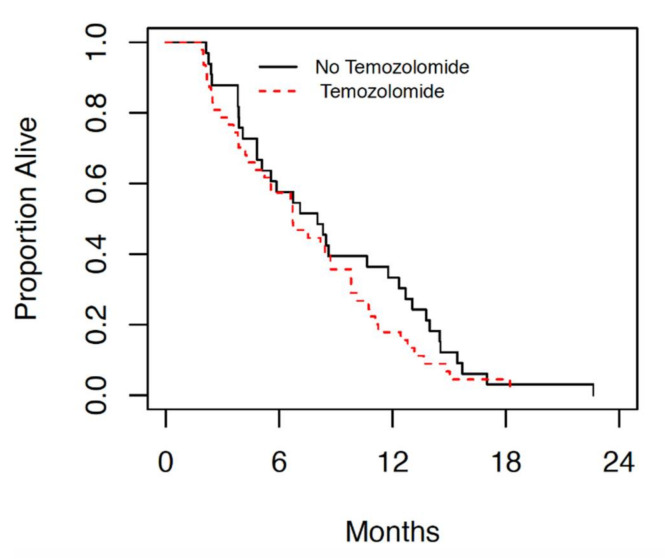
Median overall survival in patients treated with radiation alone (no temozolomide) and those who were treated with concurrent chemoradiation (temozolomide).

Amongst the patients with GBM treated at the JCC since 2014, those treated with radiation alone had a median overall survival (OS) of 8 months (4.8, 11.8 95% CI). While the median OS of those treated with radiation with concurrent and adjuvant temozolomide was 6.7 months (4.8, 8.8 95% CI), as seen in Figure 1.

**Figure 2:**
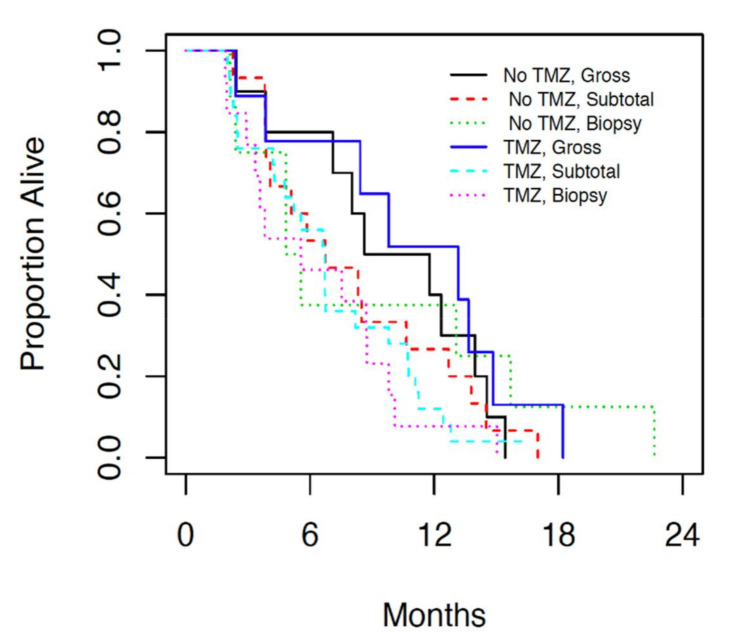
Median overall survival in patients who were treated with radiation alone (No TMZ) and chemoradiation (TMZ) subdivided further by extent of surgical resection.

Table 2 shows results of the univariable and multivariable Cox regression analyses. Only the baseline Karnofsky performance status (KPS) was statistically significant (*p* = 0.034) univariably, and after adjusting for KPS, no other factor entered the multivariable model. For every 10-point increase in KPS, the hazard ratio (95% CI) for OS decreased by 0.86 (0.75 to 0.99). This is illustrated in Figure 2, which presents the OS for patients grouped by temozolomide status and KPS (<80 versus ≥80).

**Figure 3:**
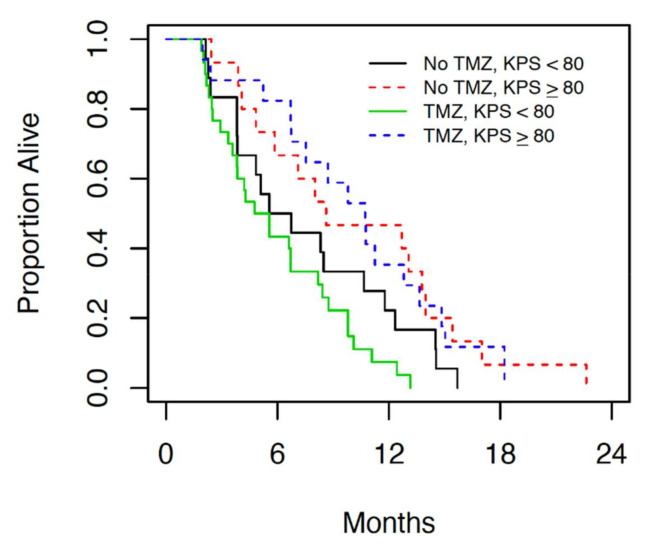
Median overall survival in patients who were treated with radiation alone (no TMZ) and chemoradiation (TMZ) subdivided further by KPS.

Analysis of patients who underwent gross total resection (>95%), subtotal resection (50–95%) and biopsy (<50%) followed by either radiation alone or radiation and concurrent temozolomide, revealed overall survival of 11.8 months (7.1, 13.7 95% CI), 6.7 months (4.8, 8.3 95% CI) and 5.6 months (3.4, 8.8 95% CI) respectively (Figure 3). No statistically significant difference was observed in terms of OS based on resection status (*p* = 0.21). However the estimated hazards ratio (0.66) for patients who underwent gross resection indicated a non-significant improved OS compared with patients who underwent biopsy alone.

**Figure 4:**
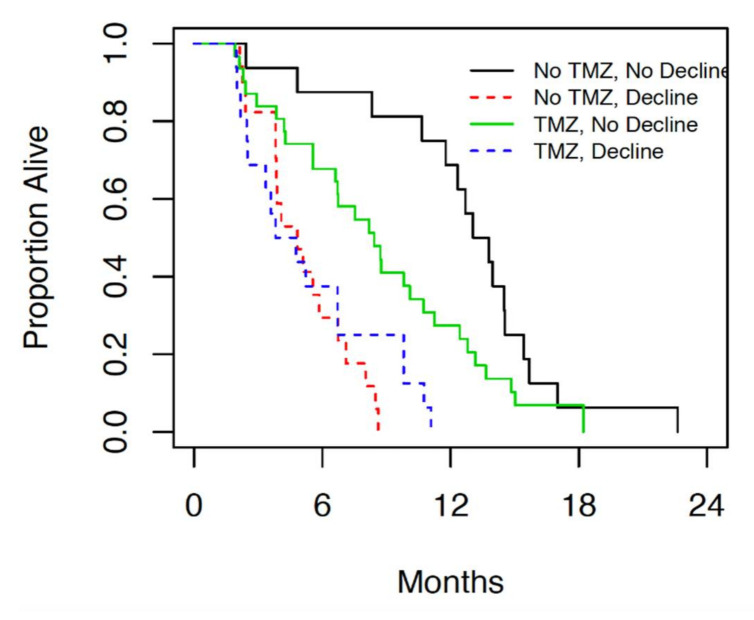
Median overall survival in patients who were treated with radiation alone (no TMZ) and chemoradiation (TMZ) subdivided further by neurologic decline post treatment.

When this patient population was compared in terms of neurologic decline following therapy, 10 or 32 (31%) of patients with KPS ≥ 80 showed neurologic decline within 1 month of treatment, while 23 of 48 (48%) patients with KPS < 80 showed neurologic decline within 1 month of treatment (Figure 4).

The incidence of neurologic decline following both radiation alone and chemoradiation was significantly higher in patients with borderline performance status compared to those with good performance status.

## 4. Discussion

Previous retrospective studies have shown that patients with borderline performance status or those aged > 65 are at a higher risk of having a poor outcome following diagnosis and treatment of GBM [9]. Our retrospective chart review demonstrates a significant difference in median overall survival in elderly patients with borderline performance status compared to those with good performance status (Figure 1 and Figure 2). The median overall survival in patients with good performance status who underwent chemoradiation is comparable to the results of the landmark trial by Perry et al., which currently provides the standard of care for GBM [5]. In patients with borderline performance status, however, OS was significantly lower than previously reported with this treatment protocol regardless of treatment modality. In patients with borderline performance status, however, no change was seen in OS with addition of TMZ. The survival in patients with borderline performance status in both treatment groups in this cohort was significantly lower than those patients in the Perry et al. study [5], suggesting a correlation between borderline performance status and poor tolerance of combined chemoradiation. The results of this retrospective review suggest that patients with borderline performance status (KPS < 80) perform poorly overall regardless of the treatment they are offered, while those with good performance status (KPS ≥ 80) respond better to combined modality treatment. This may partially be attributed to the patient’s ability to tolerate and recover from treatment side effects.

In the study by Perry et al., patients were subdivided by MGMT promoter methylation status. A significant improvement in OS was seen in the MGMT promoter methylated group. Similarly, the study by Wick et al., which compared temozolomide monotherapy and radiation alone in elderly patients, demonstrated a benefit of systemic monotherapy in MGMT promoter methylated patients [10]. In our patient population, MGMT promoter methylation status was not available for all patients. We have routinely tested for MGMT promoter methylation status since 2019, and most patients on this study were treated before 2019. Additionally, it often takes 10–15 additional business days to have these results available, and upfront treatment is often directed before this is known. Therefore, this was not further examined on our study. For future research, patients may be further subdivided by KPS and MGMT promoter methylation status to determine treatment response and outcome.

The median OS of patients who had a gross total resection compared to those who had a subtotal resection or biopsy was examined. There was not a statistically significant difference in the OS of patients based on resection status. However, there was a numerical improvement amongst patients who had a gross total resection. This difference was observed amongst patients who received chemotherapy and amongst those who did not receive chemotherapy. The lack of statistical significance may be due to small sample size, or it may be due to a true lack of effect. Further, treatment selection bias needs to be taken into account. Patients of younger age and higher KPS with tumors in the less eloquent brain are more often selected for complete resection [7]. As the extent of resection is often guided by patient factors such as KPS and age, and patient outcome is based on extent of surgical resection, which may further confound patient outcomes with adjuvant treatment. It is difficult to determine if patients with lower KPS perform poorly due entirely to their inability to tolerate adjuvant treatment or because of selection bias when receiving more extensive surgery.

The survival outcomes of patients when considering both KPS and resection status hints at the possibility of subdividing all elderly patients into two groups when considering treatment: those who have good performance status and GTR, and all others. Amongst the second group, patients can be further subdivided into those who have borderline performance status and subtotal resection or biopsy, and those who have mixed traits. This can help with further prognostication and treatment selection. In current practice at the JCC, patients who are in the first group are treated with combined modality, while those in the second group are treated with a single modality, preferably guided by MGMT promoter methylation status. In the group with mixed traits, however, the best treatment approach is often debated. In patients with mixed traits, such as those with borderline performance status with GTR or those with good performance status and STR, it may be beneficial to pursue single modality treatment rather than bimodality treatment upfront. MGMT promoter methylation status may help guide treatment with TMZ monotherapy rather than radiation monotherapy. Sparing combined modality treatment with chemoradiation in these patients can spare them significant toxicity and poor quality of life, while having no significant impact on overall survival [10]. If these patients recur, then they may potentially be treated with second line treatment with radiation or TMZ as appropriate. In these patients the clinician needs to take into account individual patient factors to make treatment decisions; however, the MGMT promoter methylation status can help with determining the role for upfront TMZ monotherapy rather than radiation monotherapy or bimodality treatment.

Neurologic decline post treatment was used as a measure of clinical outcomes in patients treated with radiation alone compared to those treated with concurrent and adjuvant chemoradiation. Though fatigue is a major side effect of this treatment, it is difficult to capture from notes alone due to its subjective nature. Patients with good performance status experienced less neurologic decline post radiation than those with borderline performance status. Neurologic decline may be confounded by the fact that location, size, and extent of resection of the tumors were not examined in relation to the incidence of neurologic decline, limiting further analysis. It should be noted that neurologic decline was seen more commonly in patients who underwent treatment with radiation alone. In these patients, temozolomide monotherapy may be an option, as the study by Wick et al. [10] showed comparable outcomes with radiation alone to temozolomide alone. MGMT promoter methylation status may be beneficial in guiding this decision in such patients.

Patient pre-existing comorbidities may also act as confounders; however, this was not examined in detail given the size and scope of this study. Specific toxicities and their effect on quality of life were not measured in this study. This may be a topic of further research. In our study we focused on neurologic decline post treatment as a measure of clinical function following treatment. This may not be a sufficient representative of patient clinical outcomes. The findings of this study are also limited by the small sample size and retrospective nature of data acquisition.

## 5. Conclusions

In the 2017 NEJM study by Perry et al. [5], the addition of temozolomide to radiation increased median overall survival from 7.6 months to 9.3 months. As a result, the treatment approach at the Juravinski Cancer Center for elderly patients with GBM shifted from short-course radiation alone to short-course radiation with concurrent and adjuvant TMZ. For the patient population at the JCC treated with short-course radiation alone compared to those treated with short-course radiation and concurrent and adjuvant TMZ, as per the NEJM study protocol, we saw a comparable median OS to those of the NEJM study. Upon further review, however, we saw a significant improvement in median OS amongst those with good baseline performance status, even compared to the previously reported values of 9.3 months [5]. In patients with borderline performance status, we saw a worse overall survival compared to the NEJM study patients, regardless of treatment modality. This brings to light the importance of taking into account the patient’s baseline KPS and comorbidities when determining the best management. In our study, patients with good performance status who underwent GTR had improved survival compared to those with borderline performance status and STR/biopsy. Combined modality treatment may not confer any additional benefit in patients with borderline performance status and STR/biopsy, while it may be beneficial in patients with good performance status and GTR. The study conducted by Wick et al. [10] demonstrates a benefit in elderly patients with MGMT promoter methylation status treated with temozolomide monotherapy. As survival in elderly patients with borderline performance status is inferior to that of patients with good performance status, there may be a role for obtaining MGMT promoter methylation status in all such patients in order to spare them the toxicity of combined therapy. This information was not available for our patients.

The patient population that we feel benefits most from our study is one with mixed traits. This includes patients with good performance status who underwent STR/biopsy or those with borderline performance status who underwent GTR. In this cohort decision making can be complex. There may be some benefit to treating these patients with single modality treatment upfront. We propose obtaining MGMT promoter methylation status in all such patients in order to determine if TMZ monotherapy or radiation monotherapy would be the ideal first step in management, thus sparing them the toxicities of combined modality treatment. If these patients progress on first line therapy, then they may be eligible for salvage TMZ or radiation as appropriate in the future. In this way, patients are spared additional toxicity while maintaining a comparable survival [10].

Overall, this study hints at performance status and extent of resection being significant determinants of patient response to treatment and prognosis. In our cohort, no clear benefit and increased toxicity was seen in patients with borderline performance status who underwent concurrent treatment with temozolomide and radiation. Bimodality treatment with chemotherapy and radiation was felt to be most helpful in patients with good performance status post gross total resection. In the case of elderly patients with borderline performance status and subtotal resection, it may be beneficial to routinely attain MGMT promoter methylation status in order to guide appropriate single modality management. In those who have mixed traits, such as borderline performance status with gross total resection or good performance status with subtotal resection, clinical judgement and MGMT promoter methylation status should be considered to determine treatment options. Ultimately, single modality treatment in patients with mixed traits with regards to performance status and surgical extent may result in preservation of quality of life while having minimal impact on survival. Further randomized studies are needed in order to substantiate this hypothesis.

## Figures and Tables

**Table 1:** Demographics table.

Statistic	Radiation Alone Results (N = 33)	Chemoradiation Results (N = 47)	*p*-Value
**Median (range) years of age**	73 (65–86)	74 (65–84)	0.28
**N (%) sex**			
Male	19 (57.6%)	24 (51.1%)	0.57
Female	14 (42.4%)	23 (48.9%)	
**N (%) KPS score**			
<80	18 (54.6%)	30 (63.3%)	0.4
≥80	15 (45.4%)	17 (36.2%)	
Median (range) KPS score	70 (40–90)	70 (40–90)	0.77
**Median (range) MMSE score**	27 (12–30)	27 (12–30)	0.8
**N (%)**			
Gross total resection	10 (30.3%)	9 (19.2%)	0.51
Subtotal resection	15 (45.4%)	25 (53.2%)	
Biopsy only	8 (24.2%)	13 (27.7%)	
**Median (range) months Duration of Temozolomide**			
Concurrent	-	0.7 (0.2, 0.8)	NC
Maintenance (*n* = 26)		4.8 (0.2, 14.8)	
Total		0.8 (0.2, 15.5)	
**N (%) Completed Radiation Treatment**	33 (100.0)	46 (97.9)	1
**Tumor Location**			
Corpus collosal	1 (3.0)	1 (2.1)	NC
Frontal	11 (33.3)	9 (19.1)	
Frontoparietal	2 (6.1)	4 (8.5)	
Frontotemporal	2 (6.1)	5 (10.6)	
Occipital	2 (6.1)	0 (0)	
Parietal	2 (6.1)	10 (21.3)	
Parietooccipital	2 (6.1)	3 (6.4)	
Temporal	8 (24.2)	10 (21.3)	
Temporoparietal	1 (3.0)	4 (8.5)	
Thalamus	2 (6.1)	1 (2.1)	
**Side**			
Both	1 (3.0)	1 (2.1)	0.82
Left	16 (48.4)	26 (55.3)	
Right	16 (48.4)	20 (42.6)	

**Table 2:** Results of the univariable and multivariable Cox regression analyses.

Variable	Comparator	N	HR (95% CI)	*p*-Value
Gender	M vs. F	80	1.16 (0.74, 1.84)	0.52
Age at Diagnosis	/year	80	0.97 (0.93, 1.02)	0.21
Karnofsky	/10 points	80	0.86 (0.75, 0.99)	0.034
MMSE	/point	75	0.97 (0.92, 1.02)	0.18
Radiation Duration	/day	80	0.92 (0.73, 1.16)	0.48
Resection Status	Gross Total Subtotal Biopsy Only	80	0.66 (0.35, 1.25) 1.10 (0.63, 1.89) Reference	0.21
Concurrent TMZ	Y vs. N	80	1.30 (0.82, 2.05)	0.27
Adjuvant TMZ	Y vs. N	80	0.78 (0.48, 1.26)	0.30
Any TMZ	Y vs. N	80	1.31 (0.83, 2.08)	0.25
Multivariable Model				
Karnofsky	/10 points	80	0.86 (0.75, 0.99)	0.034

## Data Availability

The data presented in this study are available on request from the corresponding author. The data are not publicly available due to restriction from the Hamilton Integrated Research Ethics Board as they contains specific patient information.

## Supplementary Materials

The following are available online at https://www.mdpi.com/article/10.3390/curroncol28040220/s1, Table S1: Location specific outcomes.
