# Supplementary material for: Outcomes in Elderly Patients with Glioblastoma Multiforme Treated with Short-Course Radiation Alone Compared to Short-Course Radiation and Concurrent and Adjuvant Temozolomide Based on Performance Status and Extent of Resection
Source: Curr Oncol. 2021 Jun 26;28(4):2399–408. doi: 10.3390/curroncol28040220 (PMC8293260; doi:10.3390/curroncol28040220)
Supplement: Supplementary file 1 [file curroncol-28-00220-s001.zip › curroncol-1178088-SI.pdf]

Table S1. Location specific outcomes.

Location	Location specific Outcomes
**Frontotempotal**	0/7 had GTR (0%)<br>5/7 had neurologic decline post RT (71.4%)<br>6/7 completed RT (85.7%)
**Frontoparietal**	1/6 had GTR (16.7%)<br>2/6 had neurologic decline post RT (33.3%)<br>6/6 completed RT (100%)
**Temporoparietal**	1/5 had GTR (20%)<br>2/5 had neurologic decline post RT (40%)<br>5/5 completed RT (100%)
**Frontal**	6/20 had GTR (30%)<br>11/20 had neurologic decline post RT (55%)<br>20/20 completed RT (100%)
**Corpus collosal**	0/2 had GTR (0%)<br>1/2 had neurologic decline post RT (50%)<br>2/2 completed RT (100%)
**Temporal**	6/18 had GTR (33.3%)<br>5/18 had neurologic decline post RT (27.7%)<br>18/18 completed RT (100%)
**Parietal**	3/12 had GTR (25%)<br>3/12 had neurologic decline post RT (25%)<br>12/12 completed RT (100%)
**Occipital**	0/2 had GTR (0%)<br>1/2 had neurologic decline post RT (50%)<br>2/2 completed RT (100%)
**Thalamus**	0/3 had GTR (0%)<br>2/3 had neurologic decline post RT (66.7%)<br>3/3 completed RT (100%)
**Parieto-occipital**	2/5 had GTR (40%)<br>1/5 had neurologic decline post RT (20%)<br>5/5 completed RT (100%)
